# Di­aqua­bis­[5-(2-pyrazin-2-yl)tetra­zolato]copper(II)–pyrazine-2-carbo­nitrile (1/2)

**DOI:** 10.1107/S1600536814002293

**Published:** 2014-02-05

**Authors:** Mohamed Abdellatif Bensegueni, Aouatef Cherouana, Slimane Dahaoui, Issam Boudraa

**Affiliations:** a Unité de Recherche de Chimie de l’Environnement et Moléculaire Structurale, Université Constantine 1, Algeria; b CRM2, UMR-CNRS 7036, Jean Barriol Institut, Lorraine University, BP 230, 54506 Vandoeuvre-lés-Nancy Cedex, France

## Abstract

The title compound, [Cu(C_5_H_3_N_6_)_2_(H_2_O)_2_]·2C_5_H_3_N_3_, is a 1:2 co-crystal between the mononuclear complex di­aqua­bis­[5-(pyrazin-2-yl)tetra­zolato]copper(II) and the reagent pyrazine-2-carbo­nitrile which was used in the synthesis. The Cu^II^ atom is located on an inversion centre and has a distorted octa­hedral [4 + 2]-coordination environment formed by four N atoms of two chelating bidentate 5-(pyrazin-2-yl)tetra­zolate ligands at shorter distances and two water O atoms at longer distances. The Cu^II^ complex molecules are held together by O—H⋯N hydrogen bonds and π–π stacking inter­actions [centroid–centroid distance 3.6139 (8) Å], forming layers parallel to (100). These layers alternate with layers of pyrazine-2-car­bo­nitrile mol­ecules and both are held together *via* C—H⋯N hydrogen bonds and further π–π stacking inter­actions.

## Related literature   

For related Cu^II^ complexes, see: Liu *et al.* (2007 ▶); Abu-Youssef *et al.* (2007 ▶). For hydrogen-bonding networks and IR spectroscopy of related complexes, see: Abu-Youssef *et al.* (2007 ▶). For the synthesis of the title compound, see: Zhao *et al.* (2008 ▶).
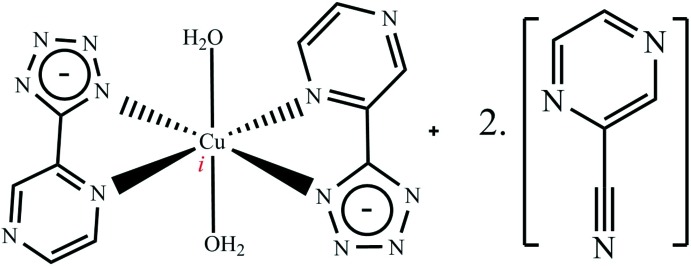


## Experimental   

### 

#### Crystal data  

[Cu(C_5_H_3_N_6_)_2_(H_2_O)_2_]·2C_5_H_3_N_3_
*M* *_r_* = 604.06
Monoclinic, 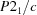

*a* = 13.591 (2) Å
*b* = 12.784 (3) Å
*c* = 7.216 (2) Å
β = 104.93 (2)°
*V* = 1211.4 (5) Å^3^
*Z* = 2
Mo *K*α radiation
μ = 0.96 mm^−1^
*T* = 100 K
0.10 × 0.08 × 0.06 mm

#### Data collection  

Agilent SuperNova CCD diffractometer
72054 measured reflections
3711 independent reflections
3265 reflections with *I* > 2σ(*I*)
*R*_int_ = 0.036

#### Refinement  

*R*[*F* ^2^ > 2σ(*F* ^2^)] = 0.027
*wR*(*F* ^2^) = 0.075
*S* = 1.08
3711 reflections
195 parameters
2 restraints
H atoms treated by a mixture of independent and constrained refinement
Δρ_max_ = 0.62 e Å^−3^
Δρ_min_ = −0.24 e Å^−3^

### 

Data collection: *CrysAlis PRO* (Oxford Diffraction, 2008 ▶); cell refinement: *CrysAlis PRO*; data reduction: *CrysAlis PRO*; program(s) used to solve structure: *SIR2002* (Burla *et al.*, 2003 ▶); program(s) used to refine structure: *SHELXL97* (Sheldrick, 2008 ▶); molecular graphics: *ORTEP-3 for Windows* (Farrugia, 2012 ▶); software used to prepare material for publication: *PLATON* (Spek, 2009 ▶).

## Supplementary Material

Crystal structure: contains datablock(s) I. DOI: 10.1107/S1600536814002293/qk2063sup1.cif

Structure factors: contains datablock(s) I. DOI: 10.1107/S1600536814002293/qk2063Isup2.hkl

CCDC reference:

Additional supporting information:  crystallographic information; 3D view; checkCIF report

## Figures and Tables

**Table 1:** Selected bond lengths (Å)

Cu1—N3	1.9853 (9)
Cu1—N2	2.0583 (10)
Cu1—O1	2.3477 (9)

**Table 2:** Hydrogen-bond geometry (Å, °)

*D*—H⋯*A*	*D*—H	H⋯*A*	*D*⋯*A*	*D*—H⋯*A*
O1—H1*W*⋯N5^i^	0.80 (2)	2.07 (2)	2.8577 (13)	170 (2)
O1—H2*W*⋯N6^ii^	0.81 (1)	2.05 (1)	2.8543 (14)	173 (2)
C1—H1⋯N9^iii^	0.95	2.57	3.2993 (17)	134
C3—H3⋯N4	0.95	2.51	3.2825 (15)	138
C11—H11⋯N1^iv^	0.95	2.51	3.2800 (17)	138

Symmetry codes: (i) 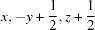; (ii) 
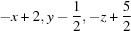
; (iii) 
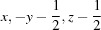
; (iv) 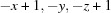.

**Fig. 1.**
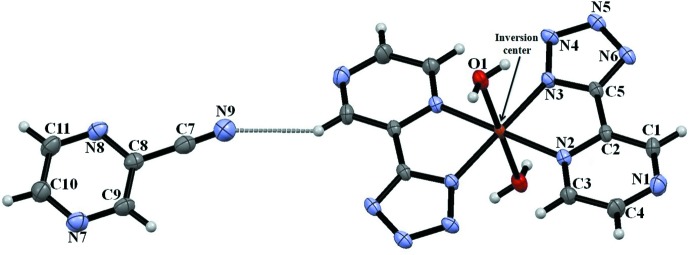
Molecular structure of the title compound [Cu(Pyztz)2(H2O)2].(CPy)2 with displacement ellipsoids drawn at the 50% probability level.

**Fig. 2.**
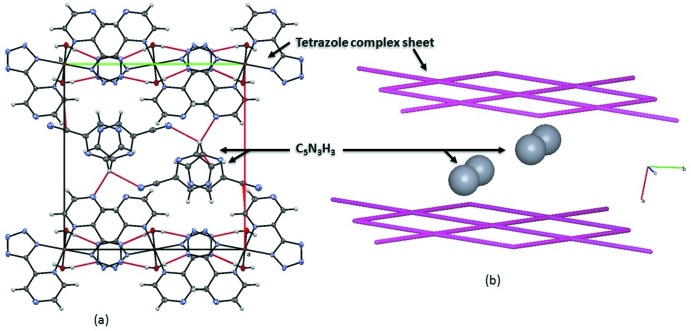
(a) The crystal structure of [Cu(Pyztz)2(H2O)2].(CPy)2 viewed along [001] showing hydrogen bonds as red lines. (b) The 4-connected sql net topology of the O—H···N hydrogen bonds in [Cu(Pyztz)2(H2O)2].(CPy)2.

**Fig. 3.**
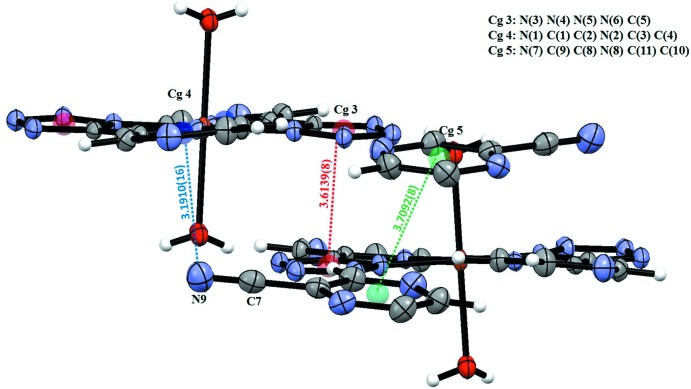
π-π stacking interactions in [Cu(Pyztz)2(H2O)2].(CPy)2 with Cg···Cg distances in Å.

## supplementary crystallographic information

### 1. Comment

In continuation of related research (Abu-Youssef *et al.*, 2007; Liu *et al.*, 2007), we set out to investigate a new copper compound with the aim to study its molecular and crystal structure including hydrogen bonding and network topology. The title compound, [Cu(Pyztz)_2_(H_2_O)_2_].(CPy)_2_ (Fig. 1), crystallizes in the monoclinic space group *P*2_1_/c, and the asymmetric unit contains an anionic 5-(pyrazin-2-yl)tetra­zolato ligand (Pyztz), one coordinated water molecule, one central Cu^2+^ ion which is located on an inversion centre, and one pyrazine-2-carbo­nitrile molecule (CPy). In the complex [Cu(Pyztz)_2_(H_2_O)_2_] the copper ion adopts a Jahn-Teller-distorted o­cta­hedral coordination by four N atoms of two chelating bidentate Pyztz ligands and by two water O atoms at distinctly longer distances (Table 1). The two Pyztz ligands of the complex are essentially coplanar and the complex is reinforced by two intra­molecular hydrogen bonds C3—H3···N4 (Table 2). Identical Cu complexes with bond lengths similar to the title compound were reported from [Cu(Pyztz)_2_(H_2_O)_2_] (Abu-Youssef *et al.*, 2007) and from [Cu(Pyztz)_2_(H_2_O)_2_].H_2_O (Liu *et al.*, 2007; Abu-Youssef *et al.*, 2007).

The [Cu(Pyztz)_2_(H_2_O)_2_] complexes in the title compound are arranged in layers parallel (100) and form two-dimensional infinite networks parallel this plane (Fig. 2). They are held together by O—H···N hydrogen bonds between the water molecules as donors and tetra­zole nitro­gen atoms N5 and N6 as acceptors (Table 2) and by π-π stacking inter­actions between pairs of tetra­zole rings (Fig. 3; Cg3···Cg3). Inserted between the layers of the [Cu(Pyztz)_2_(H_2_O)_2_] complexes are layers of CPy molecules, which are held together by π-π stacking (Fig. 3, Cg5···Cg5) and by weak inter­molecular C—H···N hydrogen bonds to N4 and N9 as acceptors (Fig. 2, Table 2).

From the topological viewpoint the structure of [Cu(Pyztz)_2_(H_2_O)_2_].(CPy)_2_ may be considered as tetra­gonal plane net, the Cu complex could be regarded as node. Each complex is bound to four other complexes and acting as 4-connected node and each cyano­pyrazine fragment is between two tetra­gonal planes net. In this way the structure could be reduced to an uninodal 4 - c net, of sql topological type, and the Point (Schlafli) symbol for the net is 4^4^ 6^2^ (Fig. 2 b).

### 2. Experimental

The compound was prepared under solvothermal conditions, by a mixture of 0.065 g (1 mmol) of sodium azide, 2 ml of pyrazine-2-carbo­nitrile and 0.255 g (1 mmol) of copper fluoborate hydrate according to a literature procedure.(Zhao *et al.*, 2008). The mixture was kept indisturbed for a few days at room temperature and furnished blue crystals suitable for X-ray diffraction.

### 3. Refinement

Carbon bonded H atoms were placed at calculated positions and were treated as riding on the parent C atoms with C—H = 0.95 Å, *U*_iso_(H) = 1.2*U*_eq_(C). The two H atoms of the water molecule were refined with distance restraints of O—H = 0.82 (1) Å and H–H = 1.40 (2) Å, whereas their *U*_iso_(H) were refined freely.

### Crystal data

**Table:**

[Cu(C_5_H_3_N_6_)_2_(H_2_O)_2_]·2C_5_H_3_N_3_	*F*(000) = 614
*M**_r_* = 604.06	*D*_x_ = 1.656 Mg m^−^^3^
Monoclinic, *P*2_1_/*c*	Mo *K*α radiation, λ = 0.71073 Å
Hall symbol: -P 2ybc	Cell parameters from 70 reflections
*a* = 13.591 (2) Å	θ = 2.2–30.6°
*b* = 12.784 (3) Å	µ = 0.96 mm^−^^1^
*c* = 7.216 (2) Å	*T* = 100 K
β = 104.93 (2)°	Prism, blue
*V* = 1211.4 (5) Å^3^	0.1 × 0.08 × 0.06 mm
*Z* = 2	

### Data collection

**Table:**

Agilent SuperNova CCD diffractometer	3265 reflections with *I* > 2σ(*I*)
Radiation source: micro-source	*R*_int_ = 0.036
Multi-layer monochromator	θ_max_ = 30.6°, θ_min_ = 2.2°
φ and ω scans	*h* = −19→19
72054 measured reflections	*k* = −16→18
3711 independent reflections	*l* = −10→10

### Refinement

**Table:**

Refinement on *F*^2^	Primary atom site location: structure-invariant direct methods
Least-squares matrix: full	Secondary atom site location: difference Fourier map
*R*[*F*^2^ > 2σ(*F*^2^)] = 0.027	Hydrogen site location: inferred from neighbouring sites
*wR*(*F*^2^) = 0.075	H atoms treated by a mixture of independent and constrained refinement
*S* = 1.08	*w* = 1/[σ^2^(*F*_o_^2^) + (0.0387*P*)^2^ + 0.609*P*] where *P* = (*F*_o_^2^ + 2*F*_c_^2^)/3
3711 reflections	(Δ/σ)_max_ = 0.001
195 parameters	Δρ_max_ = 0.62 e Å^−^^3^
2 restraints	Δρ_min_ = −0.24 e Å^−^^3^

### Special details

**Table:**

Geometry. All s.u.'s (except the s.u. in the dihedral angle between two l.s. planes) are estimated using the full covariance matrix. The cell s.u.'s are taken into account individually in the estimation of s.u.'s in distances, angles and torsion angles; correlations between s.u.'s in cell parameters are only used when they are defined by crystal symmetry. An approximate (isotropic) treatment of cell s.u.'s is used for estimating s.u.'s involving l.s. planes.
Refinement. Refinement of *F*^2^ against ALL reflections. The weighted *R*-factor *wR* and goodness of fit *S* are based on *F*^2^, conventional *R*-factors *R* are based on *F*, with *F* set to zero for negative *F*^2^. The threshold expression of *F*^2^ > 2σ(*F*^2^) is used only for calculating *R*-factors(gt) *etc*. and is not relevant to the choice of reflections for refinement. *R*-factors based on *F*^2^ are statistically about twice as large as those based on *F*, and *R*- factors based on ALL data will be even larger.

### Fractional atomic coordinates and isotropic or equivalent isotropic displacement parameters (Å^2^)

**Table:**

	*x*	*y*	*z*	*U*_iso_*/*U*_eq_	
Cu1	1.0000	0.0000	1.0000	0.01027 (6)	
O1	0.89916 (7)	−0.01043 (7)	1.21907 (13)	0.01521 (17)	
H1W	0.9008 (14)	0.0452 (12)	1.271 (3)	0.022 (4)*	
H2W	0.9197 (14)	−0.0577 (11)	1.294 (2)	0.028 (5)*	
N1	0.71720 (8)	−0.15880 (9)	0.54002 (16)	0.0195 (2)	
N2	0.87742 (8)	−0.05530 (7)	0.79219 (14)	0.01180 (18)	
N3	0.97170 (8)	0.15216 (7)	0.96694 (14)	0.01155 (18)	
N4	0.90083 (8)	0.21867 (8)	0.86795 (14)	0.01393 (19)	
N5	0.93288 (8)	0.31435 (8)	0.91544 (15)	0.01499 (19)	
N6	1.02487 (8)	0.31291 (8)	1.04561 (15)	0.01458 (19)	
N7	0.64661 (9)	0.29939 (9)	0.97445 (17)	0.0217 (2)	
N8	0.51739 (8)	0.13314 (9)	0.80887 (16)	0.0188 (2)	
N9	0.66710 (10)	−0.07623 (10)	0.97158 (19)	0.0269 (3)	
C1	0.78740 (9)	−0.21147 (9)	0.67013 (17)	0.0155 (2)	
H1	0.7816	−0.2853	0.6786	0.019*	
C2	0.86881 (9)	−0.16041 (8)	0.79360 (16)	0.0115 (2)	
C3	0.80715 (10)	−0.00197 (9)	0.66417 (17)	0.0148 (2)	
H3	0.8108	0.0722	0.6597	0.018*	
C4	0.72809 (10)	−0.05505 (10)	0.53648 (18)	0.0186 (2)	
H4	0.6802	−0.0157	0.4434	0.022*	
C5	1.04598 (9)	0.21169 (8)	1.07398 (16)	0.0116 (2)	
C7	0.64409 (10)	0.01012 (10)	0.9505 (2)	0.0195 (2)	
C8	0.61209 (9)	0.11813 (10)	0.91986 (17)	0.0165 (2)	
C9	0.67664 (10)	0.19991 (10)	1.00151 (18)	0.0191 (2)	
H9	0.7432	0.1845	1.0778	0.023*	
C10	0.55197 (10)	0.31480 (11)	0.86613 (19)	0.0214 (3)	
H10	0.5274	0.3845	0.8444	0.026*	
C11	0.48782 (10)	0.23276 (11)	0.78358 (19)	0.0210 (2)	
H11	0.4213	0.2483	0.7073	0.025*	

### Atomic displacement parameters (Å^2^)

**Table:**

	*U*^11^	*U*^22^	*U*^33^	*U*^12^	*U*^13^	*U*^23^
Cu1	0.01191 (10)	0.00595 (9)	0.01099 (10)	−0.00037 (6)	−0.00058 (7)	−0.00041 (6)
O1	0.0205 (4)	0.0105 (4)	0.0139 (4)	−0.0007 (3)	0.0032 (3)	−0.0003 (3)
N1	0.0178 (5)	0.0192 (5)	0.0187 (5)	−0.0033 (4)	0.0000 (4)	−0.0030 (4)
N2	0.0139 (4)	0.0097 (4)	0.0116 (4)	−0.0010 (3)	0.0028 (3)	−0.0009 (3)
N3	0.0138 (4)	0.0090 (4)	0.0110 (4)	0.0004 (3)	0.0016 (3)	0.0002 (3)
N4	0.0170 (5)	0.0104 (4)	0.0142 (4)	0.0030 (3)	0.0035 (4)	0.0020 (3)
N5	0.0197 (5)	0.0104 (4)	0.0155 (5)	0.0016 (4)	0.0057 (4)	0.0009 (3)
N6	0.0202 (5)	0.0089 (4)	0.0158 (4)	−0.0004 (4)	0.0068 (4)	−0.0004 (3)
N7	0.0188 (5)	0.0222 (5)	0.0225 (5)	−0.0026 (4)	0.0024 (4)	−0.0030 (4)
N8	0.0151 (5)	0.0225 (5)	0.0176 (5)	−0.0017 (4)	0.0020 (4)	−0.0032 (4)
N9	0.0231 (6)	0.0247 (6)	0.0316 (6)	−0.0003 (5)	0.0044 (5)	0.0031 (5)
C1	0.0168 (5)	0.0135 (5)	0.0154 (5)	−0.0044 (4)	0.0026 (4)	−0.0028 (4)
C2	0.0141 (5)	0.0103 (5)	0.0107 (5)	−0.0020 (4)	0.0042 (4)	−0.0015 (4)
C3	0.0159 (5)	0.0133 (5)	0.0139 (5)	0.0002 (4)	0.0014 (4)	0.0005 (4)
C4	0.0171 (6)	0.0186 (6)	0.0164 (6)	−0.0002 (4)	−0.0023 (4)	−0.0004 (4)
C5	0.0155 (5)	0.0087 (5)	0.0117 (5)	−0.0013 (4)	0.0051 (4)	−0.0004 (4)
C7	0.0153 (6)	0.0248 (6)	0.0178 (6)	−0.0019 (4)	0.0032 (4)	0.0007 (5)
C8	0.0154 (5)	0.0199 (6)	0.0141 (5)	−0.0015 (4)	0.0038 (4)	−0.0002 (4)
C9	0.0144 (5)	0.0228 (6)	0.0184 (6)	−0.0025 (4)	0.0012 (4)	−0.0009 (5)
C10	0.0196 (6)	0.0209 (6)	0.0225 (6)	0.0011 (5)	0.0033 (5)	−0.0019 (5)
C11	0.0153 (6)	0.0246 (6)	0.0207 (6)	0.0010 (5)	0.0005 (5)	−0.0030 (5)

### Geometric parameters (Å, º)

**Table:**

Cu1—N3	1.9853 (9)	N7—C10	1.3365 (17)
Cu1—N3^i^	1.9853 (9)	N8—C11	1.3338 (17)
Cu1—N2	2.0583 (10)	N8—C8	1.3431 (16)
Cu1—N2^i^	2.0583 (10)	N9—C7	1.1467 (17)
Cu1—O1	2.3477 (9)	C1—C2	1.3915 (15)
Cu1—O1^i^	2.3477 (9)	C1—H1	0.9500
O1—H1W	0.801 (17)	C2—C5^i^	1.4538 (16)
O1—H2W	0.810 (13)	C3—C4	1.3978 (17)
N1—C4	1.3355 (16)	C3—H3	0.9500
N1—C1	1.3355 (16)	C4—H4	0.9500
N2—C3	1.3325 (15)	C5—C2^i^	1.4538 (16)
N2—C2	1.3491 (14)	C7—C8	1.4475 (18)
N3—C5	1.3390 (15)	C8—C9	1.3940 (17)
N3—N4	1.3441 (13)	C9—H9	0.9500
N4—N5	1.3139 (14)	C10—C11	1.3955 (19)
N5—N6	1.3567 (15)	C10—H10	0.9500
N6—C5	1.3299 (14)	C11—H11	0.9500
N7—C9	1.3346 (18)		
			
N3—Cu1—N3^i^	180.0	C11—N8—C8	115.26 (11)
N3—Cu1—N2^i^	81.16 (4)	N1—C1—C2	121.32 (11)
N3^i^—Cu1—N2^i^	98.84 (4)	N1—C1—H1	119.3
N3—Cu1—N2	98.84 (4)	C2—C1—H1	119.3
N3^i^—Cu1—N2	81.16 (4)	N2—C2—C1	121.24 (11)
N2^i^—Cu1—N2	180.0	N2—C2—C5^i^	113.46 (10)
N3—Cu1—O1	90.45 (4)	C1—C2—C5^i^	125.21 (10)
N3^i^—Cu1—O1	89.55 (4)	N2—C3—C4	120.04 (11)
N2^i^—Cu1—O1	91.85 (4)	N2—C3—H3	120.0
N2—Cu1—O1	88.15 (4)	C4—C3—H3	120.0
N3—Cu1—O1^i^	89.55 (4)	N1—C4—C3	122.66 (11)
N3^i^—Cu1—O1^i^	90.45 (4)	N1—C4—H4	118.7
N2^i^—Cu1—O1^i^	88.15 (4)	C3—C4—H4	118.7
N2—Cu1—O1^i^	91.85 (4)	N6—C5—N3	111.32 (10)
O1—Cu1—O1^i^	180.0	N6—C5—C2^i^	130.06 (10)
Cu1—O1—H1W	108.3 (13)	N3—C5—C2^i^	118.51 (10)
Cu1—O1—H2W	109.2 (13)	N9—C7—C8	178.22 (15)
H1W—O1—H2W	112.8 (17)	N8—C8—C9	123.16 (12)
C4—N1—C1	116.84 (11)	N8—C8—C7	115.56 (11)
C3—N2—C2	117.82 (10)	C9—C8—C7	121.28 (11)
C3—N2—Cu1	129.08 (8)	N7—C9—C8	121.16 (12)
C2—N2—Cu1	113.08 (8)	N7—C9—H9	119.4
C5—N3—N4	106.12 (9)	C8—C9—H9	119.4
C5—N3—Cu1	113.18 (8)	N7—C10—C11	122.70 (13)
N4—N3—Cu1	140.70 (8)	N7—C10—H10	118.6
N5—N4—N3	107.83 (10)	C11—C10—H10	118.6
N4—N5—N6	110.64 (9)	N8—C11—C10	121.74 (12)
C5—N6—N5	104.10 (9)	N8—C11—H11	119.1
C9—N7—C10	115.97 (12)	C10—C11—H11	119.1
			
N3—Cu1—N2—C3	5.57 (11)	C3—N2—C2—C5^i^	174.67 (10)
N3^i^—Cu1—N2—C3	−174.43 (11)	Cu1—N2—C2—C5^i^	−6.64 (12)
O1—Cu1—N2—C3	95.74 (11)	N1—C1—C2—N2	2.97 (18)
O1^i^—Cu1—N2—C3	−84.25 (11)	N1—C1—C2—C5^i^	−173.49 (11)
N3—Cu1—N2—C2	−172.94 (8)	C2—N2—C3—C4	−0.28 (17)
N3^i^—Cu1—N2—C2	7.06 (8)	Cu1—N2—C3—C4	−178.73 (9)
O1—Cu1—N2—C2	−82.77 (8)	C1—N1—C4—C3	−1.44 (19)
O1^i^—Cu1—N2—C2	97.24 (8)	N2—C3—C4—N1	2.2 (2)
N2^i^—Cu1—N3—C5	6.01 (8)	N5—N6—C5—N3	0.31 (13)
N2—Cu1—N3—C5	−173.99 (8)	N5—N6—C5—C2^i^	−175.83 (11)
O1—Cu1—N3—C5	97.81 (8)	N4—N3—C5—N6	−0.29 (13)
O1^i^—Cu1—N3—C5	−82.19 (8)	Cu1—N3—C5—N6	179.06 (7)
N2^i^—Cu1—N3—N4	−174.98 (13)	N4—N3—C5—C2^i^	176.35 (10)
N2—Cu1—N3—N4	5.01 (13)	Cu1—N3—C5—C2^i^	−4.31 (13)
O1—Cu1—N3—N4	−83.19 (12)	C11—N8—C8—C9	−1.01 (18)
O1^i^—Cu1—N3—N4	96.82 (12)	C11—N8—C8—C7	179.02 (12)
C5—N3—N4—N5	0.14 (12)	C10—N7—C9—C8	0.21 (19)
Cu1—N3—N4—N5	−178.91 (9)	N8—C8—C9—N7	0.7 (2)
N3—N4—N5—N6	0.05 (13)	C7—C8—C9—N7	−179.38 (12)
N4—N5—N6—C5	−0.22 (13)	C9—N7—C10—C11	−0.7 (2)
C4—N1—C1—C2	−1.06 (18)	C8—N8—C11—C10	0.56 (19)
C3—N2—C2—C1	−2.18 (17)	N7—C10—C11—N8	0.3 (2)
Cu1—N2—C2—C1	176.51 (9)		

Symmetry code: (i) −*x*+2, −*y*, −*z*+2.

### Hydrogen-bond geometry (Å, º)

**Table:**

*D*—H···*A*	*D*—H	H···*A*	*D*···*A*	*D*—H···*A*
O1—H1*W*···N5^ii^	0.80 (2)	2.07 (2)	2.8577 (13)	170 (2)
O1—H2*W*···N6^iii^	0.81 (1)	2.05 (1)	2.8543 (14)	173 (2)
C1—H1···N9^iv^	0.95	2.57	3.2993 (17)	134
C3—H3···N4	0.95	2.51	3.2825 (15)	138
C11—H11···N1^v^	0.95	2.51	3.2800 (17)	138

Symmetry codes: (ii) *x*, −*y*+1/2, *z*+1/2; (iii) −*x*+2, *y*−1/2, −*z*+5/2; (iv) *x*, −*y*−1/2, *z*−1/2; (v) −*x*+1, −*y*, −*z*+1.
